# A commentary on ‘Organ preservation strategies after neoadjuvant chemoimmunotherapy in resectable non-small cell lung cancer: a multicenter retrospective cohort study’

**DOI:** 10.1097/JS9.0000000000001229

**Published:** 2024-03-04

**Authors:** Yuanyuan Wang, Limei Zhan, Yongchao He, Xiaoyu Liu, Shasha Sun

**Affiliations:** a Pulmonary and Critical Care Medicine; b Disinfection Supply Center; c Operating Room, Yantai Yuhuangding Hospital, Yantai; d Department of Emergency, Jinan Children’s Hospital, Jinan, Shandong, People’s Republic of China

It is with great interest that we read the article published in the *International Journal of Surgery*. Hui *et al*.^1^ performed a multicenter retrospective cohort study on patients with central-type non-small cell lung cancer (NSCLC) who received 2–4 cycles of neoadjuvant chemoimmunotherapy. The study’s findings demonstrated that for a subset of patients with central-type NSCLC who have positive safety and long-term survival, it is possible to apply organ preservation methods, such as modifying surgery following neoadjuvant chemoimmunotherapy.

NSCLC accounts for 85% of newly diagnosed instances of lung cancer, which continues to be the primary cause of cancer-related deaths globally^2^. Multidisciplinary therapy approaches, such as surgery combined with related systemic therapies, have grown popular in an effort to improve prognosis^3^. This study examines the long-term survival and safety of organ preservation techniques in patients with central-type NSCLC who underwent radical excision after neoadjuvant chemotherapy. This creative approach provides enlightening guidance and inspiration for the continued development of this area of research. However, there were numerous limits.

Bias resulted from the clinical features of NSCLC patients, not including BMI, tumor location, or sickness history. Published literature has demonstrated that postoperative complication risk was found to be significantly associated with a low BMI^4^. The possible reasons for this might be poor respiratory compliance, decreased lung volume, and longer operation time for overweight patients. Based on this experience, the most important factor in preventing these patients from developing postoperative complications is thorough and appropriate postoperative care starting on the day of surgery, including increased physical therapy, respiratory therapy, and aggressive prophylactic anticoagulant drug therapy. Additionally, published research has shown that whereas patients with tumors on the right who have surgery are linked to higher postoperative complications and mortality, patients with tumors on the left are connected with a protective effect against postoperative issues^5^. The right lung’s architectural features were the primary cause of the lung’s increased difficulty during surgery, increased alveolar volume loss, and decreased respiratory function. In general, patients with a tumor on the right may require more intraoperative care due to the high risk of postoperative complications.

Selection bias may have been created because the sample was split according to the modified surgical extent after neoadjuvant therapy. The two-center, retrospective nature of the study’s data meant that bias was unavoidably present during data collection. Future prospective multicenter studies are necessary to corroborate current observations further because the bias may potentially be influenced by the surgeons’ preferences and experience. Because only Chinese people were included in the study, results might not apply to other populations. Additional worldwide investigation is necessary to validate these findings. While proper clinical practice and treatment guidelines were followed to reduce any bias while maintaining standard patient care, bias may have also been contributed by the preoperative nursing of neoadjuvant chemotherapy. Preoperative nursing also means adequate treatment of complications of neoadjuvant chemotherapy.

To verify this result, more thorough and multicenter research is needed. To determine the optimal course of treatment, further in-depth prospective research, clinical trials, and analysis of indications and risk factors in different patient subgroups are needed.

## Ethical approval

This manuscript is a comment. Don’t need ethical approval.

## Consent

This manuscript is a comment. Don’t need patient consent.

## Sources of funding

Not applicable.

## Author contribution

Y.W. and L.Z.: study concept or design, data collection, data analysis or interpretation, and writing the paper; Y.H. and X.L.: data collection; S.S.: study concept or design, writing, and revising the paper.

## Conflicts of interest disclosure

This manuscript is a comment without conflicts of interest.

## Research registration unique identifying number (UIN)

This manuscript is a comment. Don’t need UIN.

## Guarantor

Shasha Sun.

## Data availability statement

This manuscript is a comment. Don’t need a Data availability statement. However, all the data from the current study are publicly available.

## Provenance and peer review

This manuscript is a comment without being invited.
